# Is There an Association Between Magnetic Resonance Imaging and Neurological Signs in Patients With Vertebral Osteomyelitis? A Retrospective Observational Study on 121 Patients

**DOI:** 10.1097/MD.0000000000002373

**Published:** 2016-01-22

**Authors:** Géraldine Bart, Hervé Redon, David Boutoille, Olivier Hamel, Lucie Planche, Yves Maugars, Benoit Le Goff

**Affiliations:** From the Rheumatology Unit, Hôtel-Dieu, Nantes University Hospital, Nantes, France (GB, YM, BLG); Radiology Department, IRIS, Nantes, France (HR); Infectious Diseases Unit, Hôtel-Dieu, Nantes University Hospital, Nantes, France (DB); Neurosurgery Unit, Hôtel-Dieu, Nantes University Hospital, Nantes, France (OH); and Biometrics and Biostatistic Platform, Hôtel-Dieu, Nantes University Hospital, Nantes, France (LP).

## Abstract

Neurological complications can occur in up to 51% of vertebral osteomyelitis (VO) in surgical series. The aim of our study was to estimate the frequency of neurological signs in a nonselected population of patients with VO and to assess clinical and MRI changes associated with these complications.

We reviewed medical charts of patients with VO from 2007 to 2014 in our University Hospital and their MRIs were analyzed by a radiologist blinded from clinical data. Neurological status was defined as follow: normal, minor signs (radiculalgia or sensory loss), and major signs (motor deficit and/or sphincter dysfunction).

A total of 121 patients were included. Mean age was 64.3 years. Overall, 50 patients (40%) had neurological signs, 26 were major signs (21.5%). Neurological signs were present at the time of admission in 37 patients and happened secondarily in 13 cases. MRI changes associated with major neurological signs were: Cervical involvement (*P* = 0.011), dural sac compression (*P* = 0.0012), ventral effacement of the subarachnoidal space (*P* < 0.001), compressive myelopathy (*P* = 0.006). More than 50% of the vertebral body destruction (*P* = 0.017), angular kyphosis (*P* = 0.016) partial or complete destruction of posterior arch (*P* = 0.032) were also associated with these signs. Neither epidural abscesses, multifocal lesions, loss of disk height, nor nerve roots compression were associated with major neurological signs.

Neurological signs occurred in 40% of our patients with one half being major signs. Cervical involvement, vertebral destruction, angular kyphosis, dural compression, effacement of subarachnoid space and compressive myelopathy on MRI were risk factors associated with neurological complications.

## Abstract 2

Supplemental Digital Content is available in the text

## INTRODUCTION

Vertebral osteomyelitis (VO) is an infection of the intervertebral disc and adjacent vertebral plates. It represents 2% to 4% of pyogenic osteoarticular infections, with an incidence of 2.4 to 5.3/100,000 person years.^1,2^ Usually this disease is caused by a hematogenous infection but sometimes by an invasive spine procedure with direct pathogen inoculation. It is a severe acute disease with potential neurological compromise and functional sequelae. Previous works have estimated that neurological complications occurred in 16% to 51% of the patients.^3–5^ However, definition of neurological complications varied widely between series and some of these studies only included patients selected from surgical wards. Therefore, their frequency in a nonselected population of VO remains to be determined.

To avoid these neurological complications, a strict immobilization is usually recommended in association with intravenous antibiotherapy.^6,7^ Magnetic resonance imaging (MRI) is the standard imaging technique for early diagnosis of VO.^8–10^ It is also widely used to assess prognosis and surgical indications of VO.^11,12^ This imaging modality helps to assess neurological structures compression as well as the infection's extent. Some MRI findings have already been described as associated with neurological complications (cervical spine lesions, epidural abscess, etc.). However, some questions remain unanswered. Some authors argue that presence of an epidural abscess should lead to surgery, although the link between these abscesses and neurological complications remains a matter of debate.^11,13,14^ Moreover, if MRI is currently used in traumatic spine injuries and spine tumors to assess spine instability^15,16^ no study has yet evaluated its interest to assess instability in acute VO.

The aim of our study was to evaluate the frequency of neurological complications in a large cohort of patients who were admitted for VO to hospital in medicine or surgery units. Secondary objectives were to search for MRI and clinical characteristics associated with these complications in order to identify a group of VO patients with higher risk of neurological complications.

## METHODS

### Study Design and Patients

This monocentric, retrospective, descriptive study was conducted in Nantes University Hospital, France. We selected medical charts of adult patients admitted for VO from January 1, 2007 until November 30, 2014. Under French law, this type of study does not need approval from an institutional review board, according to the French data protection authority. Data were collected and processed in a strictly anonymous manner. We reviewed medical charts of all patients with spine infection recorded by the informatics database in surgery and medicine departments (orthopedics, neurosurgery, rheumatology, infectious diseases, internal medicine, emergency and general medicine units). We used key words from the *International Classification of Diseases, Tenth Revision, Clinical Modification* (ICD-10-CM) to identify cases. We included only patients aged 18 or older with an infectious spondylodiscitis defined by typical MRI changes and: either microbiological identification of the causative agent on blood cultures or guided vertebral biopsy, or good response to the anti-infectious treatment if the causative agent was not identified. We excluded patients who had no microbiological identification of the causative agent and no improvement with antibiotics treatment, patients who had early postoperative infection (1 month or less), spine material infections, isolated epidural abscess, facet joint arthritis, isolated discitis or spondylitis. Patients who had no MRI at baseline were also excluded.

### Clinical Data Collection

Informatics medical records were reviewed for all the patients identified by the database code search. When a patient responded to the inclusion criteria, the full patient chart and MRI images were reviewed. Clinical data were collected and: demographic characteristics and associated pathologies of the patients, date of symptoms onset, of the first medical examination and diagnosis by MRI. Clinical data, biological parameters, and date of neurological signs were also collected.

Neurological status was assessed using the modified system of Frankel-American Spinal Injury Association (ASIA) scale at baseline and at the end of follow-up.^17^ Neurological signs were defined as follow: Major neurological signs if patients presented with motor deficit (upper or lower motor neuron dysfunction quoted by the ASIA scale) or sphincter dysfunction, and minor neurological signs if they had isolated radiculopathy (radicular pain or osteotendinous reflex abolition). Treatment and follow-up data were recorded: duration and type of antibiotic treatment, duration of rest and cast immobilization, and date of surgery.

### MRI Study and Definitions

**FIGURE 1:**
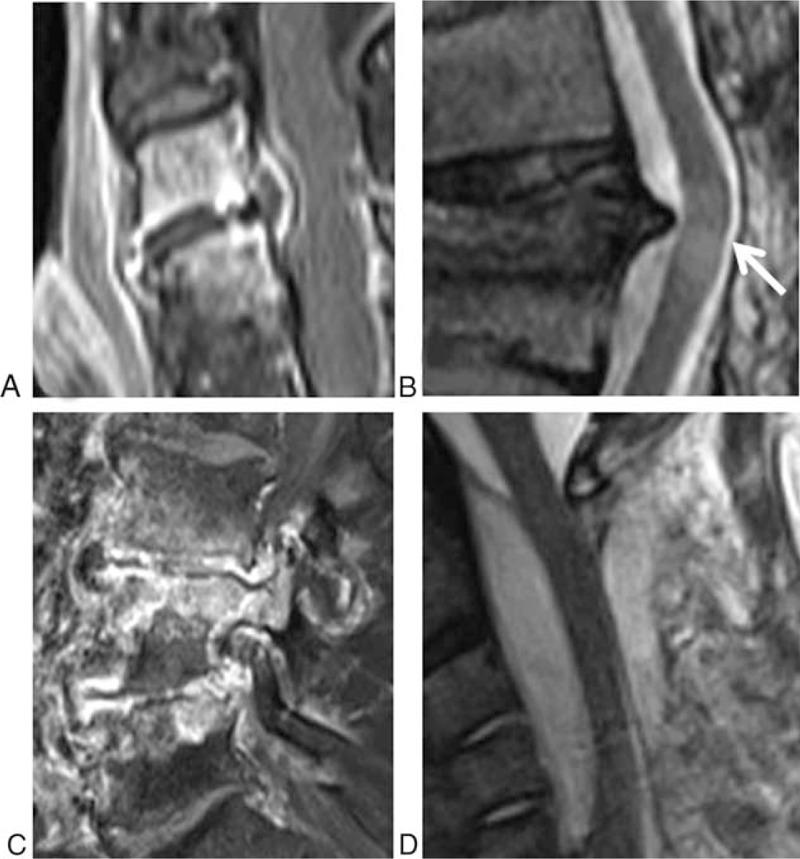
Examples of MRI abnormality: Epidural abscess in T1 weighted sagittal plane with fat saturation and gadolinium enhancement and compression of the dural sac (A), compressive myelopathy with spinal cord hypersignal (arrow) and anterior effacement of subarachnoid space in T2 weighted sagittal plane (B), edema and destruction of the posterior arch in T2 weighted sagittal plane with fat-saturation (C), voluminous epidural abscess with stenosis and anterior effacement of subarachnoid space in T2 weighted sagittal plane (D).

An independent radiologist, expert in musculoskeletal imaging, reviewed blindly all MRI acquisitions and fulfilled a standardized questioner. This questioner had been previously validated by radiologists, expert spine neurosurgeons, and rheumatologists (Figure 1).

Spondylodiscitis was defined by inflammatory changes on MRI in 2 contiguous vertebral bodies and the corresponding disk space. We reported the level involved, and noted alterations in signal intensity in the intervertebral disc or vertebral body; loss of disc height was defined by (0) normal disk height, (1) reduction <50% of normal disk height, (2) reduction of 50% to 75% of normal disk height, (3) reduction >75% of normal disk height.

Vertebral destruction was measured for the vertebrae above and below and classified as follow: (0) none, (1) <20% loss of anterior vertebral body height, (2) 20% to 50% loss of anterior vertebral body height, and (3) >50% loss of vertebral body height. Posterior arch assessment noted edema and partial or complete destruction of pedicle, facet joints, lamina and spinous process. Epidural space impairment and neurological structures compromised were also reported: canal, root, spinal cord compression, and ventral effacement of subarachnoidal space. Dural sac stenosis was assessed. Paravertebral, epidural, and disk-space abscesses were reported and measured.

We searched for instability criteria: angulation in coronal and sagittal planes was measured from the upper plate of the vertebrae above, and lower plate of the vertebrae below. We searched for posterior arch destruction and involvement of the vertebrae (corpse and/or posterior wall), as defined by Campbell et al^16^ to assess spine traumatic injuries.

If there were some missing data in the computer chart, the full paper chart was read to find out every clinical and especially neurological examination. Patients were excluded if there was no neurological examination reported in the chart.

If a patient was lost from follow-up, the last available clinical examination was considered to identify the final neurological status of each patient, and this final examination was reported as the end of follow-up date.

### Statistics

All statistical analysis was performed using SPSS software (IBM SPSS for windows, version 19.0, SPSS, Inc. Chicago, IL) for descriptive analysis and SAS software version 9.3 for univariate analysis. The Kruskal–Wallis test was used to compare continuous data and the Chi-square test or Fisher exact test was used, as appropriate, to compare categorical data. We performed univariate analysis in order to find an association between clinical or MRI characteristics and neurological signs. For all comparisons, a *P-*value < 0.05 was considered to represent a statistically significant difference. All statistical tests were 2-sided.

## RESULTS

### Patients’ Characteristics

Overall, 227 patients with spine infection were identify from the computer database research. Ninety were excluded because of the presence of spine device infections or because they had no MRI images available for reviewing. A total of 137 MRIs were reviewed, and 16 more patients were excluded because of differential diagnosis of VO (facet joint arthritis or isolated spondylitis without lesions of the intervertebral disk), or because the MRI images were too bad quality to perform a complete analysis and to fulfilled the standardized questionnaire (flow chart in Supplementary Data).

A total of 121 patients were included in our study. Main patient's characteristics are summarized in Table 1. Median duration of symptoms before diagnosis was 21.5 days (interquartile range: 9–51 days). Diagnosis was confirmed by bacteriology in 111 cases (91.7%). *Staphylococcus aureus* (33.1%) was the most frequent pathogen. Eighty-one (66.9%) patients had positive blood culture with a median duration of bacteremia of 2 days. Twenty-six (21.5%) patients had endocarditis according to the Duke Criteria.

**TABLE 1:**
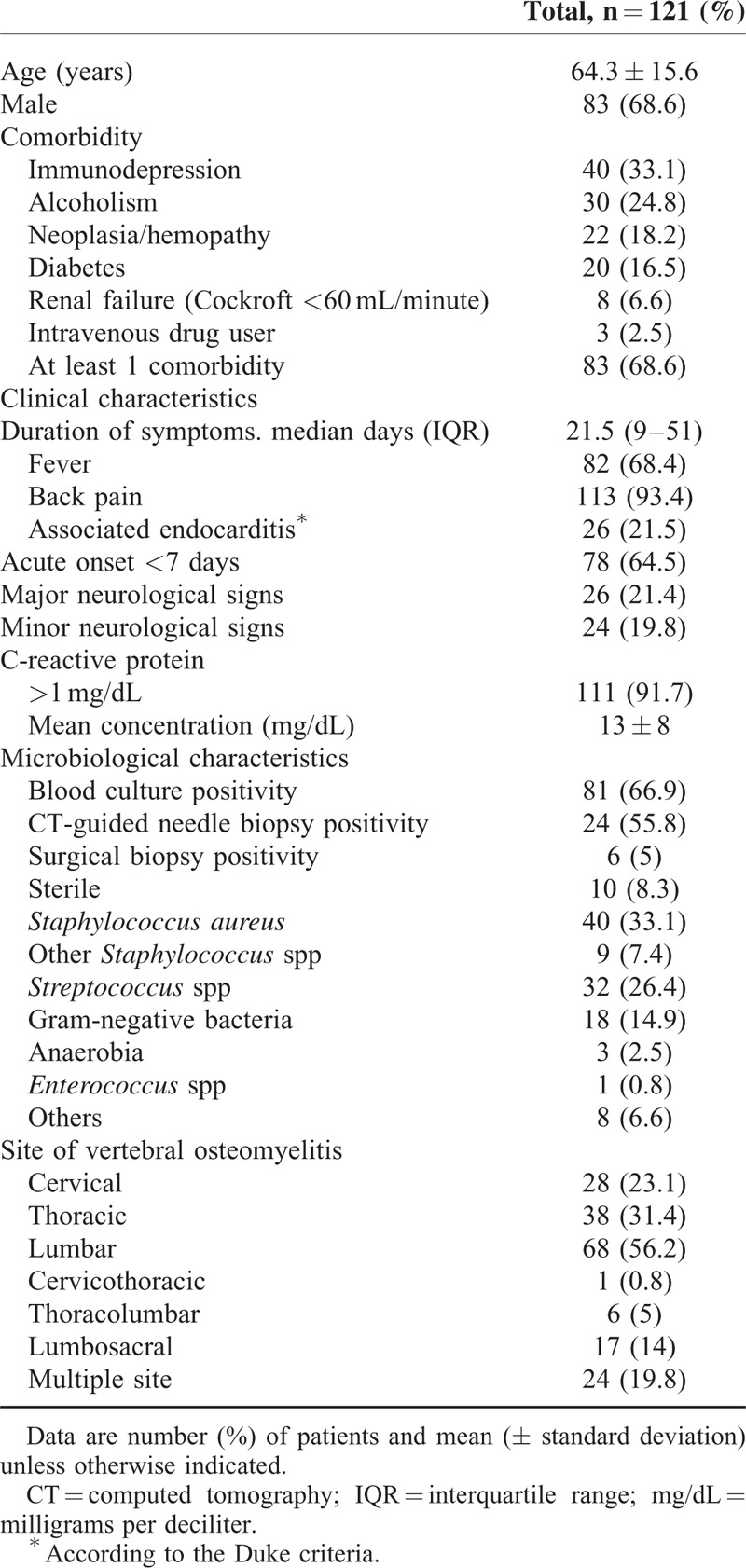
Baseline Clinical and Biological Characteristics of the 121 Patients Included in Our Study

### Neurological Evaluation

**FIGURE 2:**
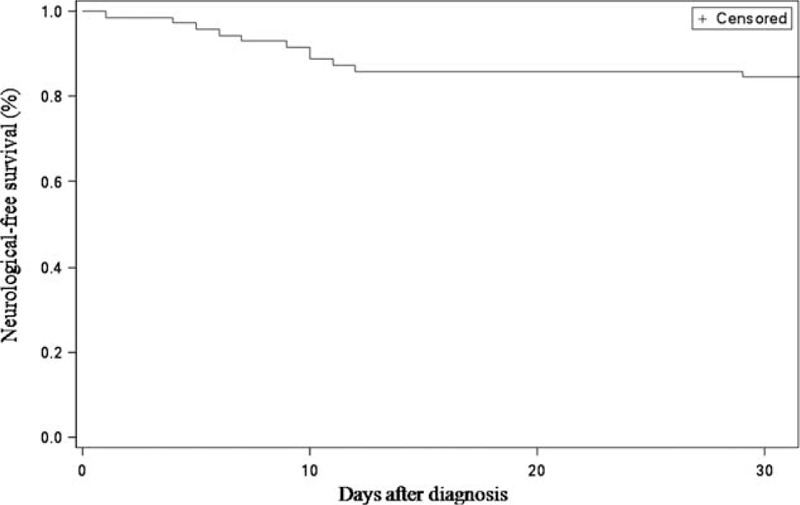
Kaplan–Meier plot showing cumulative probability of event-free survival for the 71 patients who had normal neurological examination at the time of diagnosis. Event-free survival was defined as the time period from the first medical examination to occurrence of neurological complication.

Overall, 50 patients (41.3%) had neurological signs: 26 (21.5%) had major neurological signs and 24 (19.8%) had minor ones. These signs were present at admission for 37 patients (30.6%). Thirteen (10.7%) occurred during follow-up, median time was 13.5 days after the initial diagnosis (Figure 2). Neurological signs were mainly major (84%; 11/13) when they occurred during follow-up.

According to the ASIA scale, patients with a major neurological sign were classified as followed: 8 patients had been scored D, 8 patients C, and 1 patient A. The remaining 9 patients had an ASIA score E, with isolated sphincter dysfunction.

### MRI Analysis

Median duration of symptoms before MRI diagnosis was 21.5 days (interquartile range: 9–51 days). Disk height was normal in 44.6% of patients. When it was present, loss of disk height was scored grade 1, 2, or 3 in 25.6%, 16.5%, and 13.2% of patients, respectively.

Disk abscess was present in 51.2% of patients, and homogenous or heterogeneous gadolinium enhancement of the disk was present in 11.9% and 55.1% of patients, respectively.

Regarding the vertebral inflammation and destruction, inflammation grade 3 (>50% of the vertebral corpse) was present in 68.3% of patients, while destruction grade 1, 2, or 3 were present respectively in 28.1%, 11.6%, and 5% of the patients for the vertebrae above, and in 30.6%, 12.4%, and 3.3% of the patients for the vertebrae below.

55.4% of the vertebrae above and 66.7% below had no destruction or erosions.

Partial or complete destruction of posterior arch was present in 7 patients (5.8%), and inflammation or the posterior arch was present in 95 patients (78.5%). In this case, the pediculus was more frequently affected (94 cases), followed by facet joints (74 cases), lamina (40 cases), and spinous process in 19 cases.

Epidural space was involved in 91 cases (75.2%) with an epidural phlegmon in all cases and epidural abscess in 48 patients (39.7%). Mean epidural space narrowing of the vertebral canal was 47.8% (±16.6). Seventy-three patients (60.3%) had nerve root compression, 57 (47.1%) had dural sac stenosis without myelopathy, 41 (33.9%) had anterior effacement of the subarachnoid space, and 10 (8.3%) had spinal cord hypersignal on T2-weighted sequences. Fourteen patients had abnormal angulation in sagittal view: mean value of kyphosis angle was 19.8 ± 9°, and only one patient had an abnormal angulation of 19° in frontal view. Seventy patients (57.9%) had a repeated MRI during follow-up, and 67 (55.4%) had control X-rays of spine.

### Treatment and Outcome Description

Median duration of intravenous antibiotic prescription was 16 days (interquartile range: 16–40 days), median duration of total antibiotic prescription was 86 days (interquartile range: 44–98 days). Cast immobilization was prescribed in 98 cases (80.1%) and 92 patients (76%) maintained bed rest. Median durations of cast immobilization and bed rest were 90 and 8 days, respectively.

Overall, 17 patients (14%) underwent spine surgery a median 7 days (interquartile range: 1–9) after diagnosis. Eleven patients had spinal decompression and/or stabilization, 1 patient had only surgical abscess debridement, and 5 patients had surgical abscess debridement and spinal stabilization. Among the 13 patients with secondary neurological signs, 8 underwent surgery, with a good recovery for 7 patients.

Median follow-up after admission was 191 days (interquartile range: 111–303). At the end of follow-up, 12 deaths were reported, related to the infection or treatment side-effects in 6 cases (5%). Thirty-five patients (28.9%) still complained of back pain at the end of follow-up. When performed, plain X-rays showed spine deformity in 27/67 (40.3%) of patients. Regarding neurological sequelae, 14 patients (11.5%) still had motor weakness and 10 patients had persistent radiculalgia at final examination.

### Clinical and MRI Findings Associated With Neurological Signs

In order to find clinical or MRI characteristics associated with neurological complications, we compared patients with (n = 50) and without neurological signs (n = 71) (Table 2). Clinical characteristics associated with neurological signs were an acute onset of symptoms (*P* = 0.04). There were no differences regarding the causative organism or biological findings, nor associated diseases. MRI signs associated with any neurological signs were: spinal cord hypersignal in T2-weighted sequences, dural sac stenosis, and anterior effacement of the subarachnoid space (*P* = 0.02, *P* < 0.001, and *P* < 0.001, respectively).

**TABLE 2:**
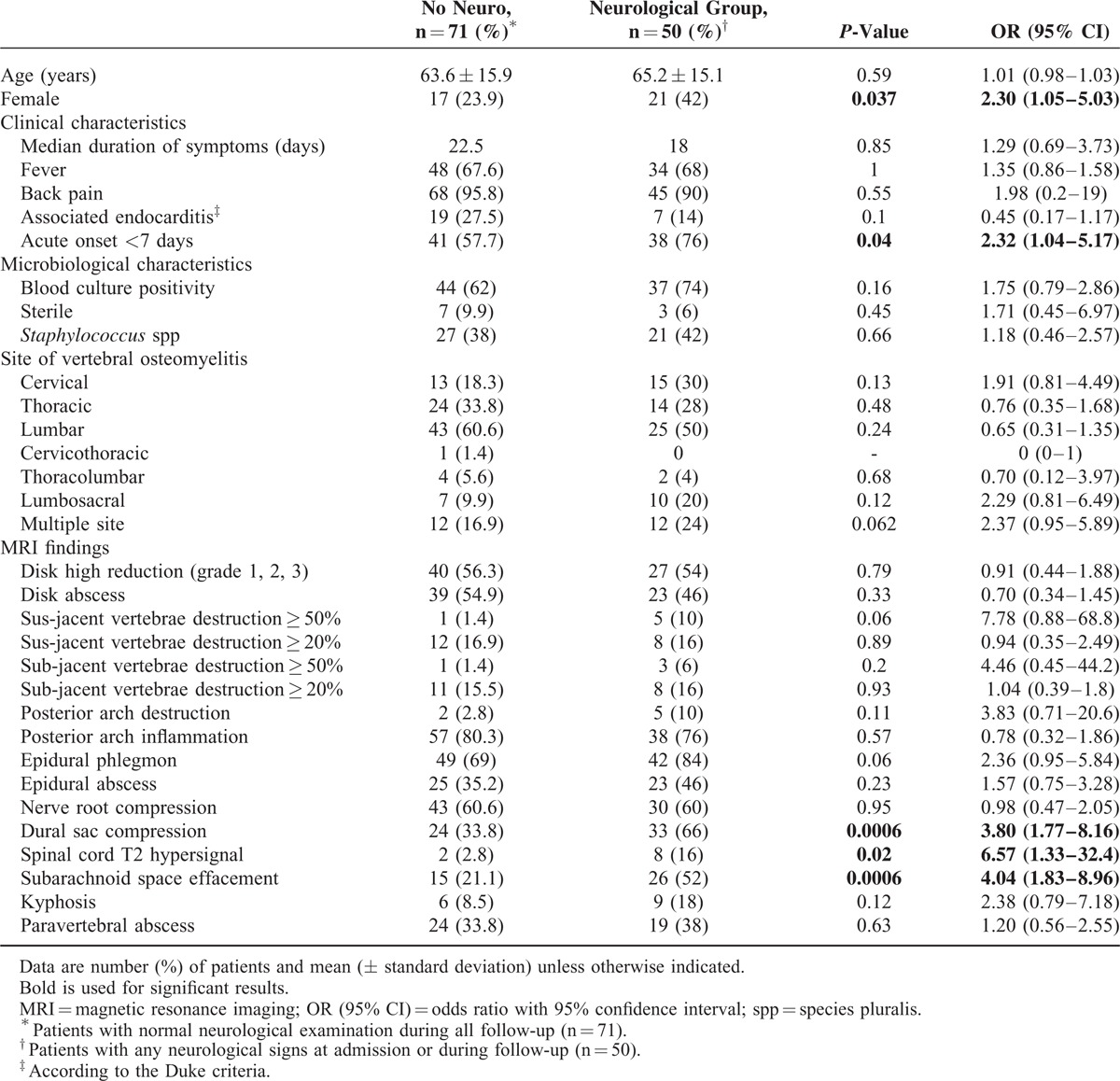
Univariate Analysis of Clinical, Microbiological, and Magnetic Resonance Imaging Criteria Associated With Neurological Signs in Patients With Vertebral Osteomyelitis

We next compared patients with major neurological signs (n = 26) to patients with normal neurological examination or minor neurological signs. As shown in Table 3, clinical characteristics associated with major neurological complications were: acute onset of symptoms (*P* = 0.005) and septicemia (*P* = 0.038). MRI signs associated with major neurological complications were: cervical lesions (*P* = 0.011), destruction grade 3 of the above vertebrae (*P* = 0.017), destruction of posterior arch (*P* = 0.032), dural sac stenosis (*P* = 0.001), spinal cord hypersignal (*P* = 0.005), and anterior effacement of the subarachnoid space (*P* < 0.001). Angular kyphosis deformity was also associated with such complications (*P* = 0.016). Nerve root compression, epidural phlegmon, and epidural abscess were not associated with major neurological complications (*P* = 0.44, *P* = 0.09, and *P* = 0.75, respectively).

**TABLE 3:**
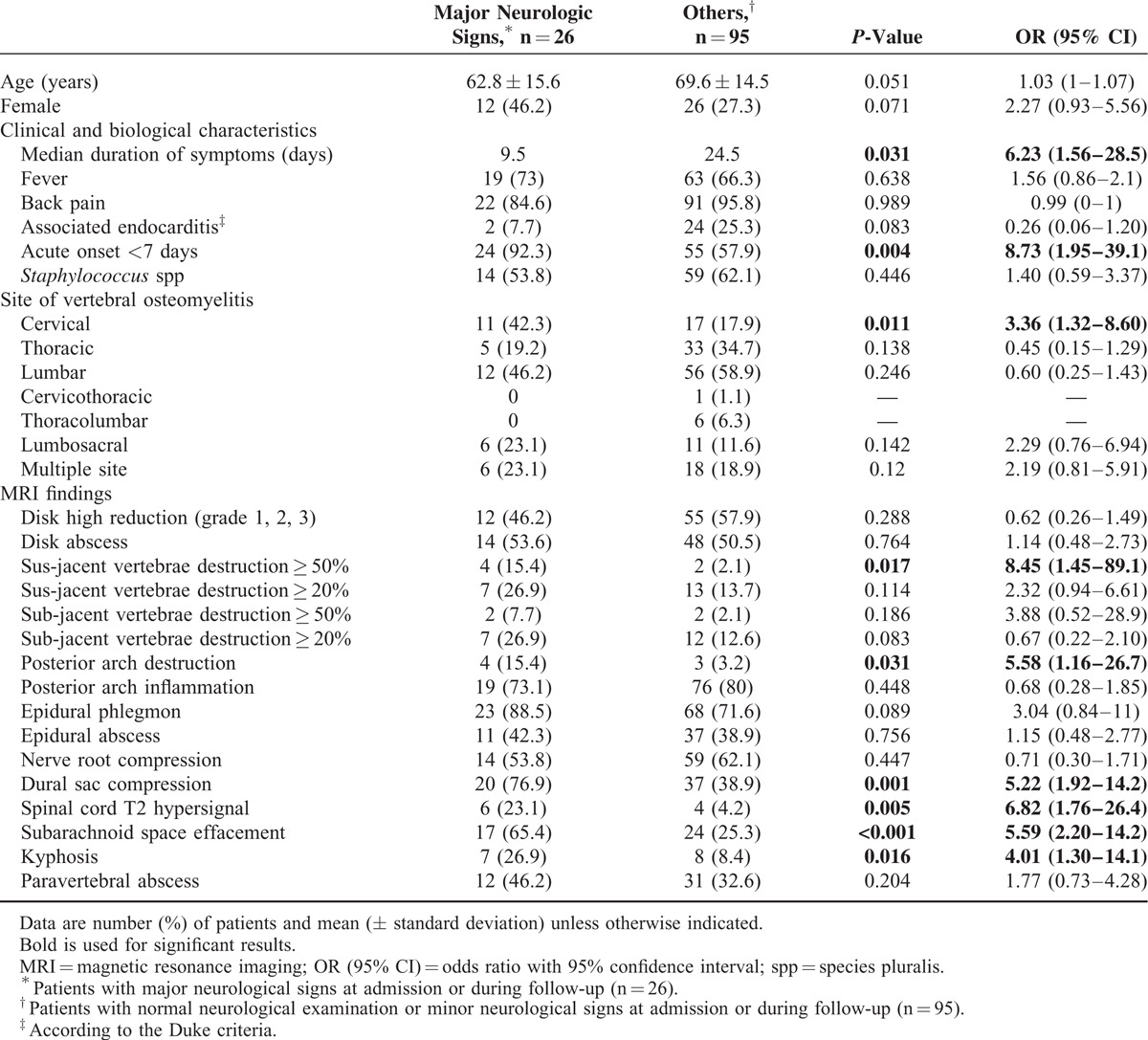
Univariate Analysis of Clinical, Microbiological, and Magnetic Resonance Imaging Criteria Associated With Major Neurological Signs in Patients With Vertebral Osteomyelitis

As shown in Table 4, we finally found that the following characteristics were associated with the occurrence of neurologic signs during follow-up: involvement of cervical spine (*P* = 0.035), destruction grade 3 of the above vertebrae (*P* = 0.044), dural sac compression (*P* = 0.008), spinal cord hypersignal (*P* = 0.003), and anterior effacement of the subarachnoid space (*P* = 0.001).

**TABLE 4:**
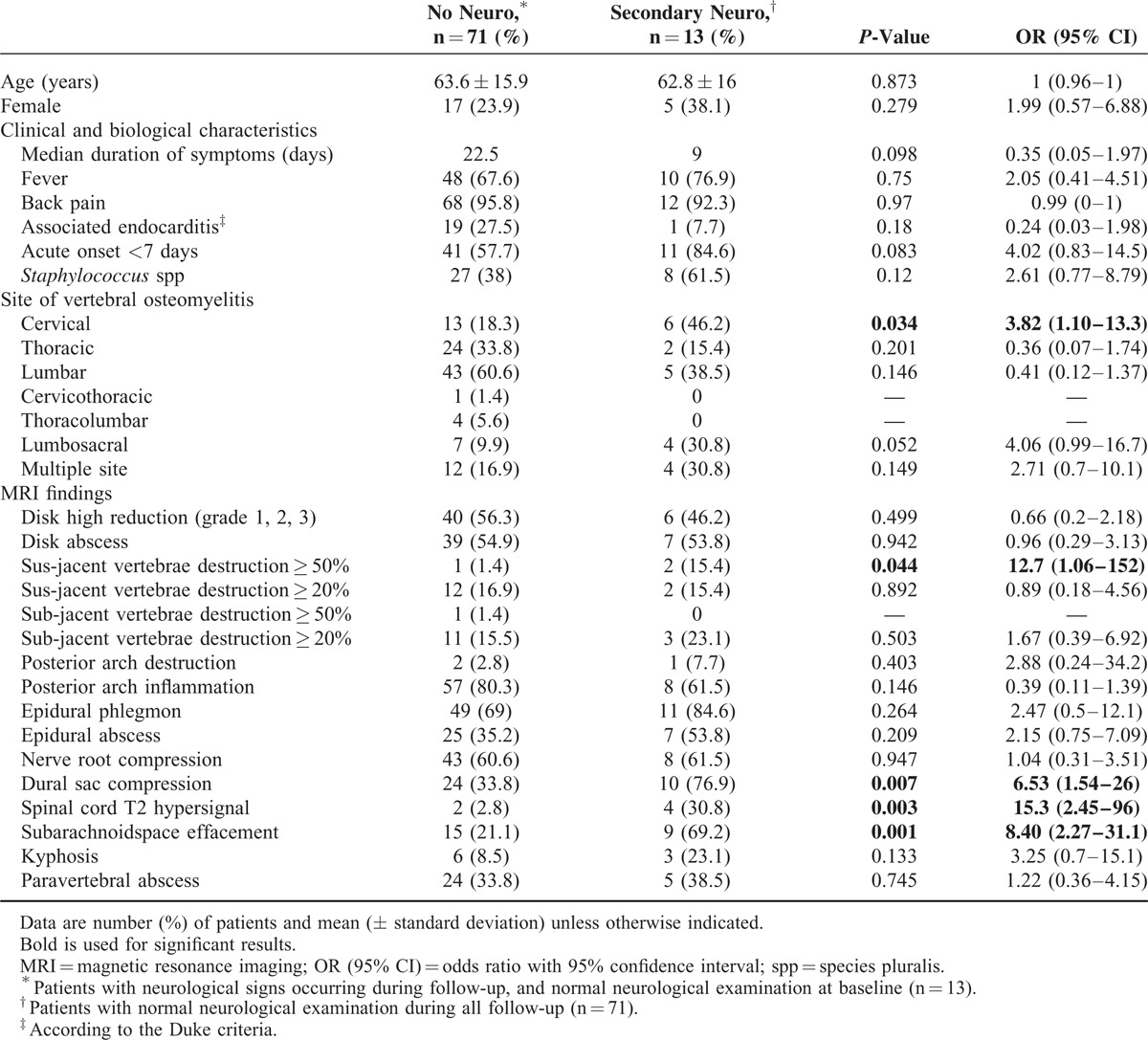
Univariate Analysis of Clinical, Microbiological, and Magnetic Resonance Imaging Criteria Associated With Secondary Neurological Signs in Patients With Vertebral Osteomyelitis

## DISCUSSION

Our study is the largest series of VO in which neurological complications and MRI imaging have been extensively studied. We have shown that neurological signs are common even in a nonselected population of VO patients. Some of the patients (10%) developed these complications during the follow-up. We described several clinical and MRI factors associated with these complications (compressive myelopathy, anterior effacement of the subarachnoid space, and destruction of the vertebral body or posterior arch). In contrast, epidural abscesses even in case of extension or reduction of the spinal canal were not associated with neurological complications.

Frequency of neurological involvement varies widely between studies, ranging from 16% to 51%.^3–5,18–22^ It even reaches 100% in case of postoperative spondylodiscitis.^18^ This variation is probably due to the lack of consensual definition of the neurological impairment across studies. Mc Henry in his large cohort of spontaneous and postoperative VO found 25% of motor deficit mainly caused by a compressive myelopathy, compressive radiculopathy, or cauda equina syndrome.^19^ In a recent large neurosurgical population of 153 patients Park et al^3^ showed 23.5% of “neurological deficit” but precisions about neurological examination were not given. Another recent study conducted by Bernard et al^5^ found 16% of neurological signs at baseline, including 5% of motor deficit and 11% of sciatica. Our study included nonselected patients from medical and surgical units. Moreover, we paid attention to report any neurological sign and classified them as minor or major according to the presence of a motor deficit or sphincter dysfunction. In our large study we totalized 40% of neurological signs with half of these signs being classified as severe.

MRI is the gold-standard imaging technique for the diagnosis of VO.^8,9^ We first found a correlation between spinal cord hypersignal and compression of the dural sac with neurological signs. These MRI signs were associated with minor as well as major neurological involvement. In contrast, nerve root compression was not associated with clinical signs. Interestingly we found that interruption of the cerebrospinal fluid even without compression of neurological structures was significantly associated with neurological complications. This sign has been described in the context of spinal stenosis^23^ but should be therefore also assessed in VO as an earlier sign of neurological compression.

Destruction of bony structures, joints, and ligaments by the infectious process can lead to direct neurological compressions and risk of instability.^24–26^ Although computed tomography scan (CT-scan) has been used as a gold standard to classify the risk of instability,^16^ MRI examination can also provide useful information since it gives additional valuable information on the ligament, disc, and neural involvement. In our work, we systematically assessed the extent of inflammation and destruction of disk, vertebrae as well as the posterior column of the spine.^27^ We found that vertebral destruction (>50%), angular kyphosis, and partial or complete destruction of posterior arch were associated with major neurological impairment. These signs were also associated with secondary neurological signs, when we assessed specifically the 13 patients who developed neurological symptoms during antibiotic course. In contrast, the importance of intervertebral space narrowing or isolated inflammation of disk and vertebral body were not associated with neurological signs. Several studies mentioned that neurological risk is related to the level of deformity or vertebral destruction, with the concept of “complicated VO.”^24–26^ Therefore, our study shows that the extent of bone destruction and the involvement of the posterior column should be assessed systematically when studying MRI images of VO to evaluate the spine stability and thus assess the risk of secondary complications.

Finally, neurological compression can be caused by compressive soft tissues: epidural or disk abscesses. In his rabbit model of VO, Feldenzer et al^28^ showed that epidural abscess volume was directly associated to neurological signs. In our standardized questionnaire, we assessed the presence of epidural phlegmon, epidural abscess, paraspinal abscess, posterior, and postero-lateral extension of infection in the epidural space and intervertebral foramina. No correlation was found between any of these signs and neurological complications. We did not even find a correlation between the level of dural sac stenosis and the presence of neurological signs. Interestingly, patients with epidural abscess were more frequently operated even without any neurological sign. Some authors have argued that epidural abscess are at high risk of instability if they are in cervical or thoracic level and should be systematically operated.^14,15^ However, surgical indication in presence of epidural abscesses still remains a matter of debate.^11,13^ Very recently, new Infection Disease Society of America (IDSA) guidelines have been published, and indications for a surgical intervention in patients with VO should be: progressive neurologic deficit, progressive deformity, spinal instability, and evidence of recurrent infection.^10^ They did not mention epidural abscess as an indication of surgery. Several studies have pointed out that epidural abscesses can disappear spontaneously with a medical treatment and were not systematically associated with neurological signs.^29,30^

Neurological complications can occur during the course of therapy. Although most of our patients had adapted back immobilization, 10% still developed neurological complication after the start of therapy. In their recent randomized controlled study, Bernard et al^5^ reported 3% of secondary neurological complication. We found that involvement of cervical spine, destruction of the vertebrae, dural sac compression, spinal cord hypersignal, and anterior effacement of the subarachnoid space were more frequent in these patients. Therefore, patients with such MRI signs but a normal baseline neurological examination should be more carefully managed. Indication of cast and immobilization is driven by pain control and prevention of neurological complications.^5^ This is sometimes difficult to endure for patients, and may generate phlebitis, bedsores, muscle loss, and autonomy disability. According to our results, neurological complications remain rare at the lumbar level, when there is no visible compression of neurological anatomical elements and limited vertebral body destruction. Indications for casting and immobilization could therefore be adapted to the initial MRI evaluation.

Our study has several limitations. This is a retrospective study with usual bias related to this kind of study design (missing data, loss to follow-up). However, we designed our study with one main objective, performed a thorough assessment of MRI images and reviewed extensively all the medical charts. Secondly, our inclusion criteria were strict, with imaging data showing an inflammation of both disc and adjacent vertebral bodies. This may have introduced selection bias with inclusion of more severe patients with extended inflammatory signs. However, clinical and microbiological characteristics were similar to other recent VO series.^5,31,32^ Finally, even with a large cohort of patients, multivariate analysis was not possible due to the low number in patients in some groups.

Despite these limitations, our study shows that in a large population of patients with VO the prevalence of neurological sign was about 41%, with 21% of patients having major neurological signs. Signs such as cervical spine involvement, extensive destruction of the vertebrae and anterior effacement of the subarachnoid space should alert on the risk of neurological complication at the initial evaluation. In contrast, epidural abscesses even in case of extension or reduction of the spinal canal were not associated with neurological complications.

## Supplementary Material

Supplemental Digital Content
