# Supplementary material for: Is There an Association Between Magnetic Resonance Imaging and Neurological Signs in Patients With Vertebral Osteomyelitis? A Retrospective Observational Study on 121 Patients
Source: Medicine (Baltimore). 2016 Jan 22;95(3):e2373. doi: 10.1097/MD.0000000000002373 (PMC4998243; doi:10.1097/MD.0000000000002373)
Supplement: Supplemental Digital Content [file medi-95-e2373-s001.pdf]

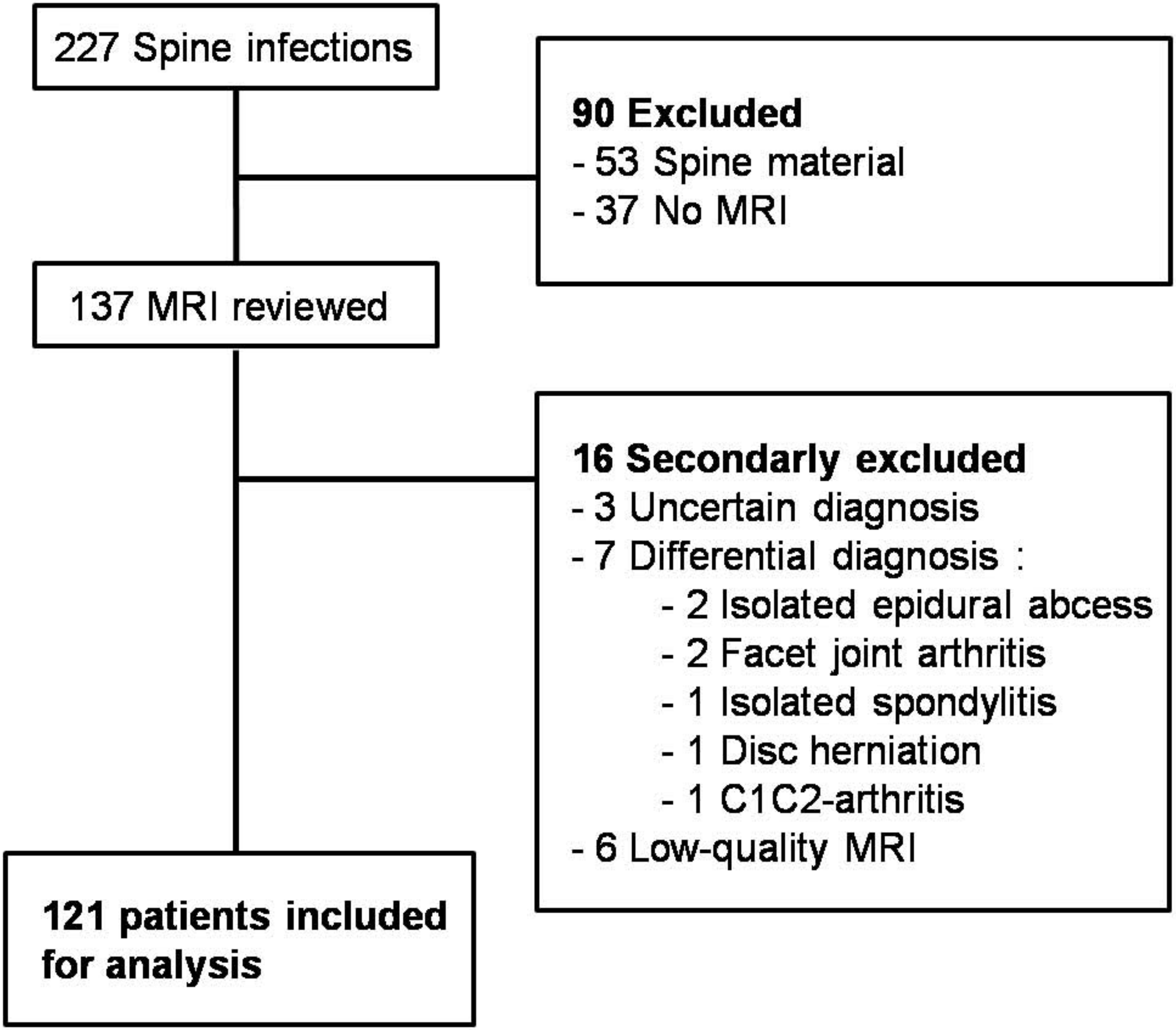
227 Spine infections
90 Excluded
- 53 Spine material
- 37 No MRI
137 MRI reviewed
16 Secondarly excluded
- 3 Uncertain diagnosis
- 7 Differential diagnosis :
- 2 Isolated epidural abcess
- 2 Facet joint arthritis
- 1 Isolated spondylitis
- 1 Disc herniation
- 1 C1C2-arthritis
- 6 Low-quality MRI
121 patients included for analysis
